# P-1794. Real-World Utility of Multiple Advanced Molecular Diagnostic Modalities at an Academic Medical Center

**DOI:** 10.1093/ofid/ofaf695.1963

**Published:** 2026-01-11

**Authors:** Sean Harford, Zoe Weiss, Eliezer Zachary Nussbaum, Majd Alsoubani, Maureen Campion

**Affiliations:** Hartford Healthcare Medical Group, Glastonbury, CT; Tufts Medical Center, Cambridge, Massachusetts; Tufts Medical Center, Cambridge, Massachusetts; Tufts Medical Center, Cambridge, Massachusetts; Tufts Medical Center, Cambridge, Massachusetts

## Abstract

**Background:**

Advanced molecular testing for pathogen identification has become a crucial part of the diagnostic toolkit for infectious disease (ID) physicians; however, several modalities and techniques are available. At our institution, we have access to broad-range PCR with reflex to Next-Generation Sequencing (NGS) (University of Washington, UW), multiplex PCR followed by targeted NGS sequencing (MicroGenDx), and microbial cell-free DNA NGS from plasma (Karius). The aim of this study was to evaluate the clinical utility of advanced molecular testing.

**Figure:**
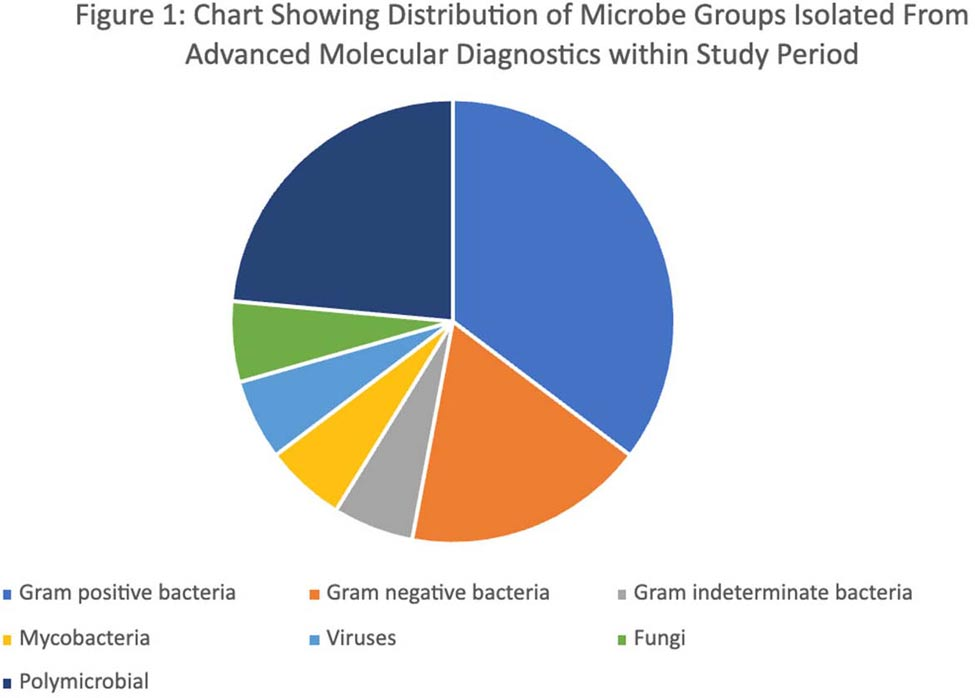

**Table 2:**
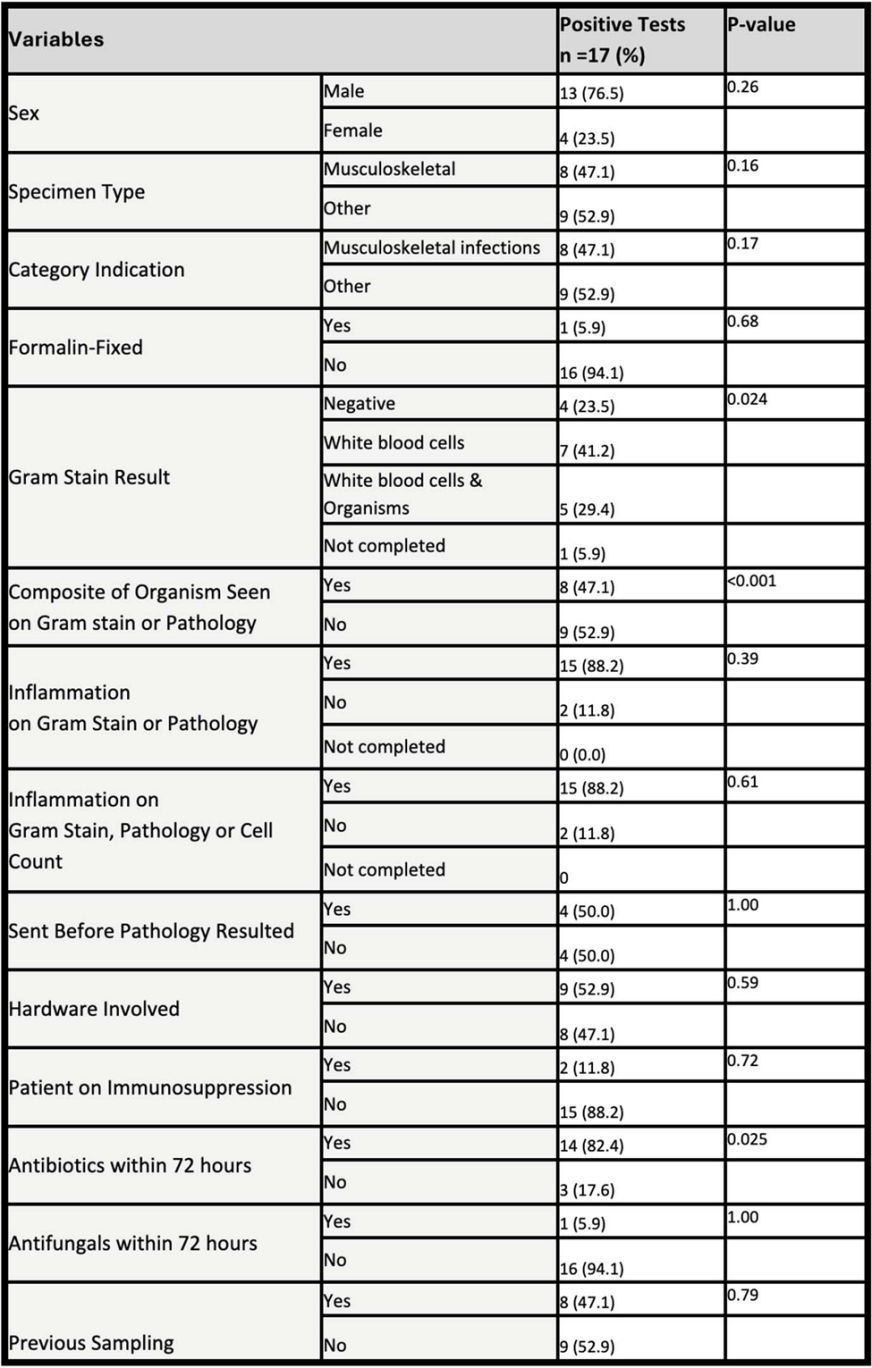
Variables Associated with Positive Advanced Molecular Diagnostic Tests

**Methods:**

We performed a single-center retrospective chart review of our institution’s usage of the above diagnostic techniques between Apr 2022 to Dec 2023. Three separate ID physicians, blinded from results, evaluated the perceived appropriateness of each request. Clinical impact (positive, neutral or negative) on care was adjudicated. Demographics and clinical data were collected. Categorical variables were summarized as counts and frequencies; continuous variables as medians with interquartile ranges. We assessed group differences using Chi-square and Mann-Whitney tests.

**Table 3:**
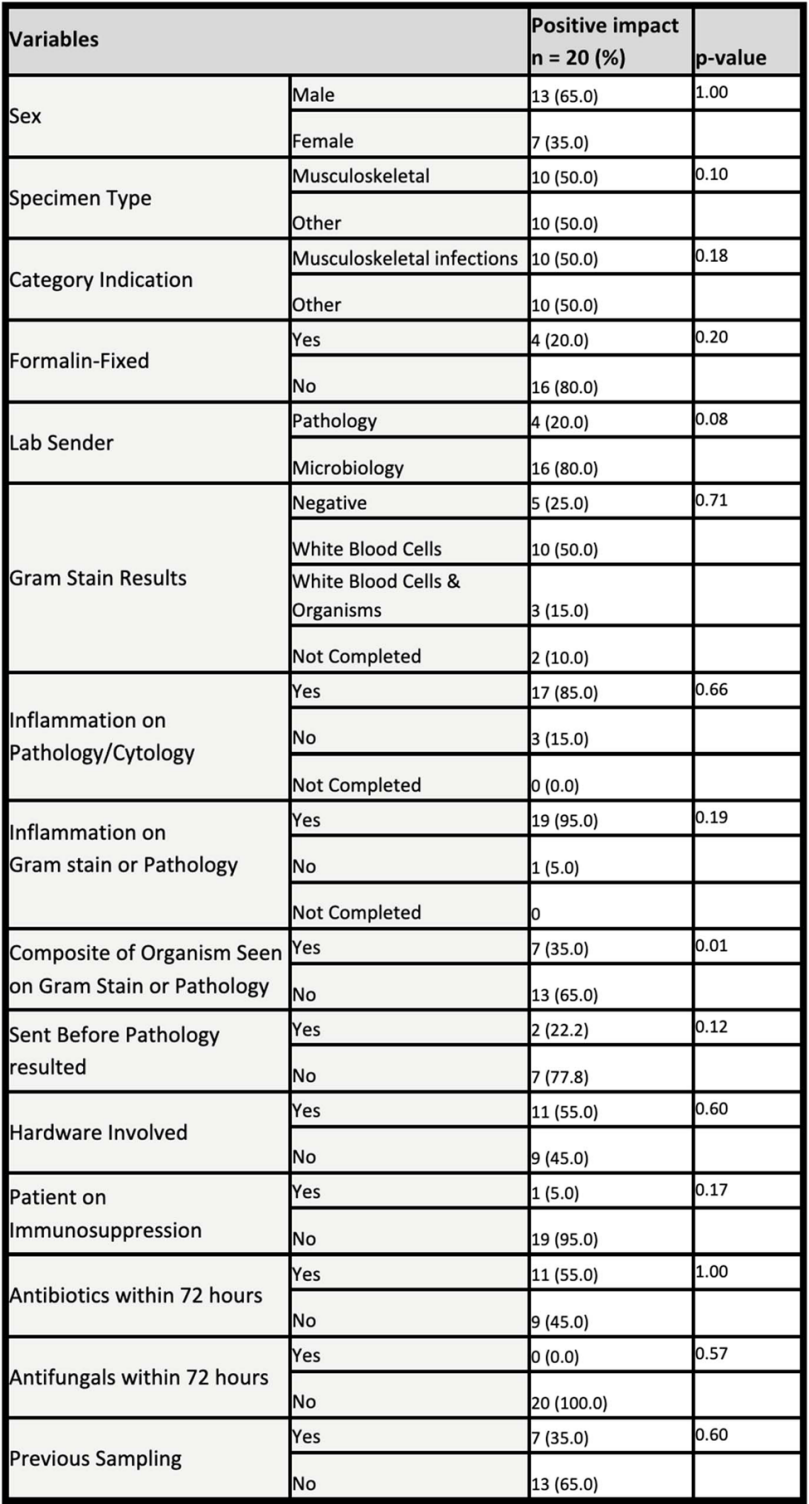
Variables Associated with Positive Impact on Clinical Care

**Results:**

We identified 76 samples for analysis, most of which were sent to UW (59%) with the rest sent to MicroGenDx (36%) and Karius (4%). Results from 17 (22%) samples were positive with one or more organisms with most (76%) of these concordant with a clinical syndrome. Figure 1 shows detected microbe groups. 80% of tests were deemed appropriate by ID physician consensus.

Including negative and positive tests, most (62%) results had a neutral clinical impact and only 29% had a positive impact. A positive test was associated with being concordant with a clinical syndrome (p< 0.001) and positive impact on clinical care (p< 0.001). Gram stain with white blood cells and organisms, positive microbiological stain on pathology and receiving antibiotics within 72 hours were significantly associated with a positive result (Table 2). Positive gram stain/microbiology stain was significantly associated with positive impact (Table 3).

**Conclusion:**

Although numerous commercially available technologies are accessible to identify causative pathogens, there is need to refine test selection criteria and develop evidence-based guidelines to maximize clinical benefit.

**Disclosures:**

Zoe Weiss, MD, Alnylam Pharmaceuticals: Stocks/Bonds (Public Company)|Anvil Diagnostics: Advisor/Consultant|Anvil Diagnostics: Ownership Interest|Cartography: Stocks/Bonds (Private Company)|Denali Therapeutics: Stocks/Bonds (Public Company)|Indomo: Stocks/Bonds (Private Company)|Maze Therapeutics: Stocks/Bonds (Public Company)

